# YTH N^6^-methyladenosine RNA Binding Protein 1 Inhibits Smooth Muscle Cell Phenotypic Modulation and Neointimal Hyperplasia

**DOI:** 10.3390/cells14030160

**Published:** 2025-01-22

**Authors:** Kai Tian, Dunpeng Cai, Shuang Yang, Wen Zhao, Xiaohan Mei, Shi-You Chen

**Affiliations:** 1 Department of Surgery, School of Medicine, University of Missouri, Columbia, MO 65212, USA; tiankai.nano@gmail.com (K.T.); dccfn@health.missouri.edu (D.C.);; 2 Department of Physiology & Pharmacology, University of Georgia, Athens, GA 30602, USA; shuang.yang@uga.edu; 3 The Research Service, Harry S. Truman Memorial Veterans Hospital, Columbia, MO 65201, USA

**Keywords:** m6A modification, smooth muscle, phenotypic modulation, YTHDF1, neointima formation

## Abstract

Smooth muscle cell (SMC) phenotypic transition contributes to several major vascular diseases such as intimal hyperplasia and restenosis, atherosclerosis, and aneurysm. However, the molecular mechanisms underlying this process are not fully understood. The objectives of this study are to determine the role of mRNA N^6^-methyladenosine (m6A) modification in SMC phenotypic modulation and injury-induced neointima formation. By using an m6A quantification kit, we found that m6A levels are altered during the early stage of SMC phenotypic modulation. RNA sequencing revealed that m6A modifications in the mRNAs of 708 genes are elevated while modifications in the mRNAs of 300 genes are decreased. These modifications occur in genes widely distributed in most chromosomes and involved in many cellular processes and signaling/gene regulations. Meanwhile, the regulators for m6A modifications are altered by PDGF-BB, a known factor inducing SMC phenotypic modulation. Although m6A writers and erasers are not altered during SMC phenotypic modulation, m6A reader YTHDF1 is dramatically reduced as early as 12 h following PDGF-BB treatment, a time much earlier than the downregulation of SMC contractile proteins. Importantly, the overexpression of YTHDF1 reverses the expression of SMC contractile proteins, suggesting a restoration of contractile SMC phenotype. By using a rat carotid artery balloon-injury model, we found that injury significantly decreases YTHDF1 levels in the medial SMCs while inducing neointima formation. Of significance, restoring YTHDF1 expression through lentiviral transduction blocks injury-induced neointima formation. Moreover, YTHDF1 delivery restores the expression of SMC contractile proteins that is diminished in arterial media layers due to the injury. These data demonstrate that YTHDF1 plays a protective role in maintaining the contractile SMC phenotype and vascular homeostasis during injury-induced pathological vascular remodeling.

## 1. Introduction

Vascular smooth muscle cells (SMCs), a major component of the arterial wall, play a critical role in arterial remodeling. The phenotypic modulation of SMCs from a contractile state to a synthetic state enables SMCs to acquire proliferative and migratory capabilities [1,2]. The SMC phenotypic transition contributes to several major vascular diseases, including intimal hyperplasia and restenosis, aneurysm formation, and atherosclerosis, especially the lesion development and fibrous cap formation [3]. The apoptosis of synthetic SMCs in fibrous cap leads to vulnerable plaque, which can cause severe cardiovascular events once ruptured, such as stroke, myocardial infarction, and sudden death. However, molecular mechanisms governing this important cellular process, especially the post-transcriptional regulation, remain largely unknown.

N6-methyladenine (m6A) mRNA modification is the most prevalent and reversible posttranscriptional RNA modification in eukaryotes. There are three types of proteins involved in regulating the levels and functions of m6A modification in RNA, including (1) “writers”, e.g., the large methyltransferase holo complex with a methyltransferase-like (METTL) 3-METTL14 catalytic core that transfers the methyl group S-adenosyl-l-methionine (AdoMet) to the N6-position of adenosines within the DRACH motif of RNA; (2) “erasers”, e.g., the fat mass- and obesity-associated protein (FTO) and AlkB homolog 5 (ALKBH5) RNA demethylase, which demethylate m6A and generate different major products: N6-hydroxymethyl adenosine (hm6A) and adenosine (A); and (3) “readers”, such as the YTH protein family, which recognize and bind m6A modification and affect the fate of the RNA. m6A influences gene expression by regulating transcription, splicing, polyadenylation, nuclear export, translation, and degradation. Recent studies have shown that m6A modification is associated with many physiological and pathological processes in cardiovascular diseases, such as atherosclerosis [4], hypertension [5], cardiac hypertrophy, and heart failure [6], and coronary heart disease [7]. Thus, m6A modification may serve as a potential target for developing therapeutics for cardiovascular diseases.

Given the important roles of SMC phenotypic modulation in various vascular diseases that are associated with m6A modification, we hypothesized that m6A is involved in the regulation of SMC phenotypic modulation. Indeed, we found that the overall m6A level is time-dependently altered during SMC phenotypic modulation. One of the m6A modification readers, YTHDF1, is significantly downregulated in the transition of SMCs from a contractile to a synthetic phenotype. However, the overexpression of YTHDF1 in SMCs can reverse the SMC phenotypic alteration. More importantly, in vivo transduction of lentivirus expressing YTHDF1 in endothelium-denuded arteries efficiently attenuates injury-induced neointimal formation. These data demonstrate that m6A modification is a novel mechanism regulating SMC phenotypic modulation, and the m6A reader YTHDF1 plays an important role in maintaining SMC homeostasis.

## 2. Materials and Methods

### 2.1. Cell Culture

Primary human SMCs were purchased from Lonza Bioscience, Catalog #: CC-2571, Walkersville, MD, USA. Rat SMCs were cultured by the enzymatic digestion method from rat carotid arteries as described previously [8]. Briefly, under sterile conditions, anesthetized Sprague–Dawley rats were placed in the supine position. A median sternotomy was performed to open the chest, and the heart was exposed. The right atrium was incised, and the left ventricle was cannulated for perfusion with sterile saline. The carotid arteries were removed and transferred to a culture dish with cold Dulbecco’s Modified Eagle Medium (DMEM) medium. After removal of the surrounding fat tissues, the arteries were longitudinally cut and placed in another cell culture dish containing DMEM. The adventitial layers were removed by straining the arteries oppositely with straight and angled forceps. The media layers were then cut into approximately 1 mm squares and transferred into cell culture plates where the arteries were digested with 1 mg/mL collagenase II (Sigma, C6885, St. Louis, MO, USA) and 100 μg/mL elastase (Sigma, E7885) at 37 °C for 25 min. The digested arteries were passed through a 70 μm strainer and washed with 5 mL of DMEM containing 10% fetal bovine serum (FBS). Endothelial cells were then removed by incubation with CD31 magnetic beads (Invitrogen, 11155D, Pittsburgh, PA, USA) following the manufacturer’s instructions. The media SMCs were plated onto a 60 mm dish and cultured with DMEM containing 20% FBS and 5% L-glutamine (Corning, Corning, NY, USA) at 37 °C in a humidified atmosphere with 5% CO_2_. One hour later, the medium with SMCs was moved to a new 60 mm dish to discard the fibroblasts attached to the original dish. After 5 days of incubation, the primary SMCs were passaged, and the phenotype of the cells was confirmed by examining smooth muscle α-actin (ACTA2) and SM22α (TAGLN) expression.

### 2.2. m6A Level Quantification

Total RNA was isolated from rat SMCs by TRIzol reagent lysis followed by 2-propanol precipitation. The m6A level was detected and quantified by a colorimetric ELISA-like assay using the commercial m6A RNA Methylation Quantification Kit (Colorimetric) (Abcam, ab185912, Cambridge, UK) based on the manufacturer’s instructions. Briefly, 200 ng RNA was bound to strip wells using the RNA high-binding solution provided in the kit. m6A was then detected using a specific N^6^-methyladenosine capture antibody and a detection antibody. The detected signal was enhanced and then quantified colorimetrically by reading the absorbance at a wavelength of 450 nm in a microplate spectrophotometer (Molecular Devices SpectraMax^®^ iD3, San Jose, CA, USA). The percentage of m6A RNA methylation in total was calculated using the formula below:

$$m6A\%=\frac{\left(\mathrm{Sample}\;\mathrm{OD}\;-\;\mathrm{NC}\;\mathrm{OD}\right)\;÷\;S}{\left(\mathrm{PC}\;\mathrm{OD}\;-\;\mathrm{NC}\;\mathrm{OD}\right)\;÷\;P}\times 100\%$$

where S and P are the amounts of the input sample and the positive control (PC) RNA (m6A oligos), respectively. NC stands for negative control (RNA containing no m^6^A).

### 2.3. m6A-Specific RNA Sequencing and Bioinformatics Analysis

Primary human SMCs were treated with vehicle (H_2_O) or PDGF-BB (10 ng/mL) for 12 h. Total RNAs were extracted using Trizol reagents (Thermofisher Scientific, Walsham, MI, USA). RNA concentrations were measured using a NanoDrop ND-1000 (Thermofisher Scientific, Waltham, MA, USA). m6A RNA library construction, sequencing, and bioinformatics analysis were performed by Arraystar Inc. (Rockville, MD, USA). Briefly, intact mRNAs were isolated from total RNA samples and chemically fragmented to around 100 nt in size. Methylated RNA immunoprecipitation (MeRIP) was performed to enrich m6A methylated mRNA fragments with an anti-m6A antibody. The RNA-seq libraries for both m6A antibody-immunoprecipitated RNAs and input RNAs were prepared with a KAPA Stranded mRNA-seq Kit (Roche, Indianapolis, IN, USA). The barcoded libraries were denatured to single-stranded DNAs, captured on Illumina flow cells, amplified in situ as sequencing clusters, and then sequenced for 150 cycles on the Illumina HiSeq 4000 system (Illumina, San Diego, CA, USA) according to the manufacturer’s instructions. Sequencer image analyses and base calling were performed using the Solexa pipeline v1.8 (Off-Line Base Caller software, v1.8). Sequencing read quality was examined by FastQC software (version 1.1). The clean reads (pass Illumina quality filter and adapter trimmed by Trimmomatic (version 0.36) were aligned to the Ensembl reference genome using HISAT2 software (v2.1.0). Differentially methylated m6A peaks compared between groups were identified by exomePeak (v1.0.0). The MeRIP-enriched regions (m6A peaks) in the RNAs were annotated with their overlapped host genes using the latest human Ensembl database.

### 2.4. Electroporation

The SNAP-tagged YTHDF1 and YTHDF2 overexpression plasmids were purchased from Addgene (Cat# 155346 and 155347, Watertown, MA, USA) [9]. The YTHDF1, YTHDF2, or control (pcDNA) plasmids were electroporated into rat SMCs by using Neon Transfection System (Thermo Fisher Scientific, MPK5000, Waltham, MA, USA) according to the manufacturer’s instruction. Briefly, SMCs were grown to 80% confluency and then digested with trypsin and collected by centrifuging. The cells were washed with PBS without Ca^2+^ and Mg^2+^ to remove cations. Then, the cells were resuspended in commercial R buffer to 1 × 10^7^ cells/mL. In total, 1~1.5 µg plasmids were mixed with 1 × 10^6^ cells. The electroporation condition for rat SMCs was 1600 V and 20 ms with 2 pulses. The cells were incubated in 10% DMEM and treated with PDGF-BB after 24 h.

### 2.5. Western Blot Analysis

After the treatment of PDGF-BB (R&D Systems, catalog # 220-BB-050, Minneapolis, MN, USA), Western blots were performed as described previously [10]. Cultured SMCs were collected and lysed by radioimmunoprecipitation assay (RIPA) lysis buffer (50 mmol/L Tris-HCI, pH 7.4, 1% Triton X-100, 0.25% wt/vol sodium deoxycholate, 150 mmol/L NaCl, 1 mmol/L EGTA, 0.1% SDS). The protein concentration was determined using the BCA Protein Assay Reagent. The proteins were then resolved in 4–20% SDS polyacrylamide gel and transferred onto nitrocellulose membranes (Bio-Rad, Hercules, CA, USA). The membranes were blocked with 5% BSA for 1 h at room temperature (RT) and then incubated with a primary antibody at room temperature for 1 h or 4 °C overnight, followed by washing with PBS (5 min, 3 times). The membranes were then incubated with IRDye secondary antibodies (LI-COR Biosciences, Lincoln, NE, USA) at room temperature for 1 h, followed by washing with PBS. The protein band was detected and quantified by the Odyssey CLx Imaging System (LI-COR Biosciences, Lincoln, NE, USA). Primary antibodies against YTHDF1 (Proteintech, 17479-1-AP, Rosemont, IL, USA), YTHDF2 (Proteintech, 24744-1-AP, Rosemont, IL, USA), FTO (Cell signaling technology, 14386S, Danvers, MA, USA), METTL3 (Proteintech, 15073-1-AP, Rosemont, IL, USA), ACTA2 (Sigma-Aldrich, A2547, St. Louis, MO, USA), Calponin 1 (Abcam, Ab22073, Cambridge, UK), SM-22α (Abcam, Ab14106, Cambridge, UK), or GAPDH (Proteintech, 10494-1-AP, Rosemont, IL, USA) were used for immunoblotting.

### 2.6. Lentivirus Package

The lentiviruses were packed in HEK-293T cells by CaCl_2_ transfection [11]. Briefly, HEK-293T cells were cultured in DMEM with 10% FBS to 60%~70% confluency in a 15 cm dish. The culture medium was changed to DMEM without FBS and incubated for 30 min. Meanwhile, pLX301-YTHDF1 or pLX301-eGFP (control) plasmids were mixed with pMD2.G, pMDLg/pRRE, and pRSV-ReV plasmids (the 3rd generation package system) at a ratio of 6:4:4.8:4.8 (in pmol) in 1.25 mL of 2× BES buffer, 312.5 μL of 1M CaCl_2_ with water supplemented to 2.5 mL. The plasmid mixture was then added to the HEK-293T cells. FBS was added to the cells to 10% (*v*/*v*) after 2 h incubation with FBS-free DMEM. After overnight incubation, the medium was changed to DMEM with 10% FBS and incubated for 2 days. The supernatant was then collected and filtered with a 0.45 μm filter following centrifugation. The lentiviruses were purified by ultracentrifugation through 2 mL 20% sucrose (*w*/*v* in PBS) at 26,000 rpm for two hours. The viruses were resuspended in PBS. A 10-fold serial dilution of viral stock was added to the wells with 2 × 10^4^ cells in a 24-well plate, and the titers of the virus were determined by counting the transduced cells with GFP expression by fluorescence microscopy. The titer of the lentivirus is 2 × 10^6^ TU/mL. The viral stock was stored at −80 °C.

### 2.7. Animals

Male Sprague–Dawley rats weighing 450 to 500 g were purchased from Charles River Laboratories (Wilmington, MA, USA). All animals were housed under conventional conditions in the University of Missouri animal care facility and received humane care in compliance with the Principles of Laboratory Animal Care formulated by the National Society for Medical Research and the Guide for the Care and Use of Laboratory Animals. Animal surgical procedures were approved by the Institutional Animal Care and Use Committee of the University of Missouri. Sex is a critical biological variable in cardiovascular diseases [12]. Since this is the first report investigating YTHDF1 function in vascular diseases, only male animals were used in this study. However, we will investigate whether there is a sex-specific role of YTHDF1 in neointima formation by including both male and female animals in our future studies.

### 2.8. Rat Carotid Artery Injury Model and Lentivirus Transduction

The rat carotid artery balloon-injury model was generated as described previously [13]. Briefly, rats were anesthetized through isoflurane inhalation. Following the incision on the left neck area and a small cut on the left external carotid artery, a 2F Fogarty arterial embolectomy balloon catheter (Baxter Edwards Healthcare, Irvine, CA, USA) was inserted into the left external carotid artery and advanced around 4 cm toward the thoracic aorta into the left common carotid artery. The balloon was then inflated with sterile and warm saline (20 µL) and then withdrawn back to carotid bifurcation with perpetual turning during the withdrawal and advancement. This process was repeated 2 extra times to ensure complete endothelial denudation. For lentiviral transduction, following the carotid balloon injury, a viral solution containing Lenti-GFP or Lenti-YTHDF1 was infused into the temporarily ligated segment of the left common carotid artery for 30 min to transduce the lentiviral vector and express YTHDF1 in medial SMCs. Fourteen days later, the rats were sacrificed, and the common carotid artery segments were isolated and removed, fixed with 4% paraformaldehyde, and then embedded in paraffin, followed by morphometric and histological analyses. Right common carotid arteries from the same rats were used as controls.

### 2.9. Histomorphometric Analyses and Immunohistochemistry (IHC) Staining

The carotid arteries were sectioned and stained as described previously [14]. Briefly, the control and injured arteries were serially cut to 5 μm tissue sections. In total, 10 sections evenly distributed in the vessel were used for staining with hematoxylin and eosin (H&E) or Elastica van Gieson reagents. The stained images were captured by a Nikon microscope (Nikon America Inc., Melville, NY, USA), and the neointima area, intima/media, and intima/lumen ratios were analyzed by ImageJ (V1.53u) [15]. The neointima formation in Lenti-YTHDF1 and Lenti-eGFP-treated arteries was compared to an uninjured control to calculate the fold change. For IHC staining, the sections were boiled in a citrate buffer (pH 6.0, Abcam, ab93678) in a microwave oven for 10 min for antigen retrieval. Then, the sections were treated with 0.3% hydrogen peroxide for 10 min followed by permeabilizing with 0.3% Triton X-100 in PBS for 30 min and blocking with 5% goat serum for 1 h. The sections were then incubated with YTHDF1, ACTA2, or calponin (CNN1) antibody (1:100) overnight followed by incubation with HRP-conjugated secondary antibody for 1 h. 3,3′-Diaminobenzidine was used to visualize the protein staining, and the sections were counterstained with hematoxylin. The images were captured with the Nikon microscope and analyzed by ImageJ software (V1.53u). The mean staining intensity was acquired from 10 different artery sections. The relative protein levels were quantified by the following formula: ([Mean value of IHC staining intensity in injured vessels minus background signal]/[mean value of IHC staining intensity of uninjured vessels minus background signal]).

### 2.10. Statistical Analysis

In vitro cell experiments were performed with at least three independent experiments and at least 10^6^ cells were used in each experiment. At least 5 animals were used in each in vivo experiment. All data were expressed as the mean ± SEM or SD. GraphPad Prism 9 was used for statistical analyses. For comparisons of two groups, a nonparametric Mann–Whitney two-tailed test was used for groups with *n* less than 6. For more than two groups, the Kruskal–Wallis test with Dunn’s multiple comparisons test for groups with *n* less than 6. Differences with *p* < 0.05 were considered significant.

## 3. Results

### 3.1. m6A Modification Is Altered During PDGF-BB-Induced SMC Phenotypic Modulation

**Figure 1:**
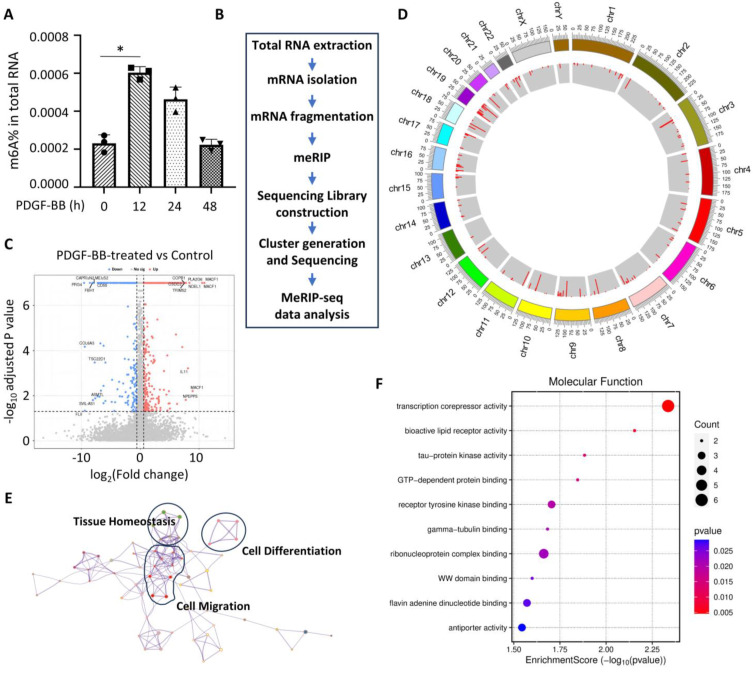
PDGF-BB induces extensive m6A modifications during SMC phenotypic modulation. (**A**) m6A modification was increased in SMCs during the early time of PDGF-BB treatment. * *p* = 0.0020 vs. vehicle-treated cells (0 h), *n* = 3. (**B**) Schematic of m6A RNA sequencing procedure. meRIP: methylated RNA immunoprecipitation. (**C**) Volcano plot of the differentially methylated genes in SMCs treated with PDGF-BB for 12 h as compared to the vehicle-treated group (Control). The blue and red dots represent statistically significant down- and up-methylated genes with a ≥1.5-fold change and statistical significance (*p* < 0.05). The top 10 up- and down-methylated genes are annotated. (**D**) Circos plot showing the distribution of enriched methylated genes on chromosomes in SMCs by PDGF-BB treatment. All chromosomes are arranged in a circle. The inner circle shows the number of differentially methylated regions. Peak heights with red color indicate significantly increased RNA methylation. The outer circle indicates the locations of the methylation sites located on each chromosome. (**E**) Metascape network of functionally enriched m6A methylated genes. Annotations denote the important processes involved in SMC phenotype modulation. (**F**) Gene Ontology—Molecular Function analyses with a bubble plot of m6A methylated genes enriched by PDGF-BB treatment, showing the functional activities important for SMC phenotype modulation.

To test whether m6A modification is involved in SMC phenotypic modulation, we first examined the m6A level in PDGF-BB-treated human aortic SMCs. Total RNA was extracted from SMCs treated with PDGF-BB for 0, 12, 24, and 48 h, respectively. The m6A level was quantified with the m6A RNA Methylation Quantification Kit (Abcam, ab185912, Cambridge, UK), which is based on a colorimetric ELISA-like assay using an m6A antibody. As shown in Figure 1A, the m6A level was elevated 2.59 times in the first 12 h of PDGF-BB treatment as compared to the vehicle-treated cells. Interestingly, the m6A levels started to decline after 24 h and gradually recovered to the original level after 48 h of PDGF-BB treatment (Figure 1A), suggesting that the m6A modification may be an initial event during SMC phenotypic modulation and thus may be involved in early determination of the phenotype alteration.

To examine the global impact of m6A modifications on SMC phenotypic modulation, we performed m6A-specific RNA sequencing (Figure 1B) in control and PDGF-treated human aortic SMCs and found that m6A modifications are increased in 708 genes while being decreased in 300 genes (Figure 1C). These genes with m6A modifications were distributed in all chromosomes (Figure 1D). Importantly, many of these genes are involved in tissue homeostasis, cell differentiation, and cell migration (Figure 1E), which are important for cell phenotype transformation. Moreover, molecular function analyses suggest that m6A modifications are important for transcription repression and other signaling-related activities (Figure 1F), consistent with the modulation of the SMC phenotype when the cells were treated with PDGF-BB. These data suggest that m6A modification is a crucial process during the SMC phenotypic modulation.

### 3.2. PDGF-BB Regulates the Expression of Genes Involved in m6A Modification

**Figure 2:**
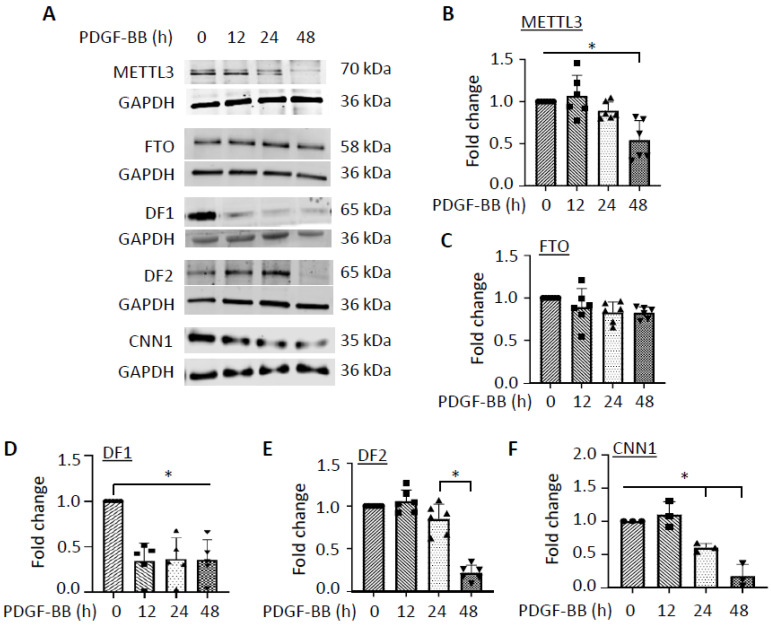
PDGF-BB regulates the expression of m6A modification regulators during SMC phenotypic modulation. (**A**) PDGF-BB downregulated the expression of m6A readers YTHDF1 (DF1) and YTHDF2 (DF2) but had no significant impact on the m6A writer METTL3 or the eraser FTO, as assessed by Western blot. (**B**–**F**) Quantification of METTL3 (**B**), FTO (**C**), DF1 (**D**), DF2 (**E**), and CNN1 (**F**) protein levels shown in (**A**) by normalizing to the corresponding GAPDH levels, respectively. * *p* < 0.01 for each comparison, *n* = 3–6.

m6A modifications are tightly controlled cellular processes involving many different protein regulators, including readers, writers, and erasers. To determine which process or regulator is essential for SMC phenotypic modulation, we examined the expression of well-characterized m6A regulators such as METTL3 (writer), FTO (eraser), and YTHDF1 and YTHDF2 (readers) during the SMC phenotype switch (Figure 2A). To make the in vitro studies relevant to the subsequent in vivo functional studies in animal models, we used rat aortic SMCs to examine the expression of m6A regulators. Interestingly, we found that the writer METTL3 and eraser FTO were not altered in rat SMCs during the early PDGF-BB treatment (Figure 2A–C). METTL3 was decreased 48 h after PDGF-BB treatment (Figure 2B). However, the m6A readers YTHDF1 and YTHDF2 appeared to be dramatically downregulated by PDGF-BB (Figure 2A,D–F). Importantly, YTHDF1 expression was significantly decreased as early as 12 h after the PDGF-BB treatment, prior to the downregulation of the SMC marker protein (Figure 2A,D–F), suggesting a potentially functional role in SMC phenotypic modulation. Like METTL3, YTHDF2 expression was not downregulated until 48 h after PDGF-BB treatment, indicating that METTL3 and YTHDF2 may not be involved in the initiation of SMC phenotypic modulation although it may be involved in the subsequent cellular events such as SMC proliferation.

### 3.3. Overexpression of YTHDF1 Restores SMC Contractile Protein Expression Downregulated During SMC Phenotypic Modulation

**Figure 3:**
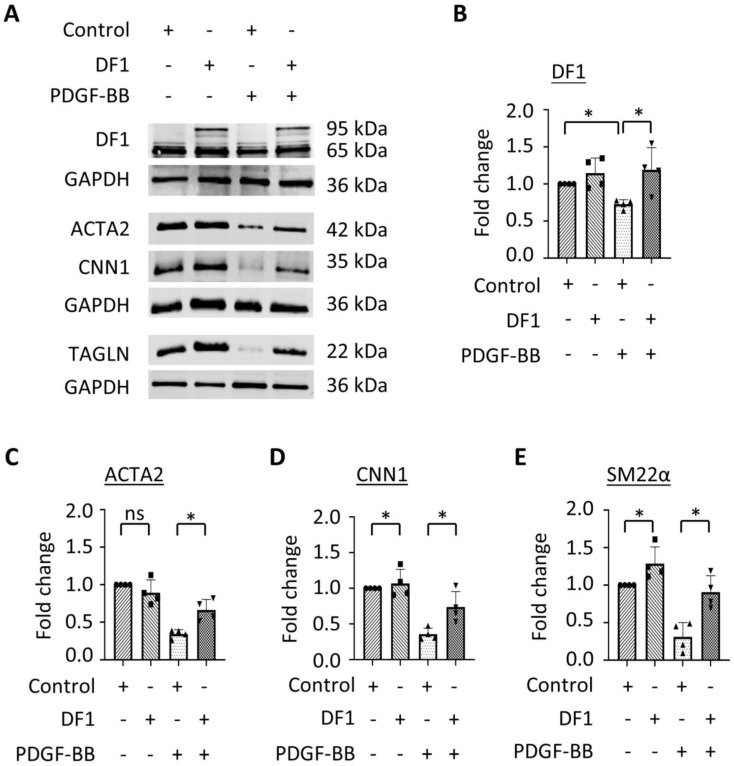
YTHDF1 negatively regulates SMC phenotypic modulation. SMCs were transfected with control vector or YTHDF1 (DF1) plasmid via electroporation followed by PDGF-BB treatment for 48 h. (**A**) Overexpression of YTHDF1 restored SMC marker protein expression downregulated by PDGF-BB, as assessed by Western blot analyses. (**B**–**E**) Quantification of the protein levels shown in A by normalizing to the corresponding GAPDH levels, respectively. Both exogenous and endogenous YTHDF1 proteins were included in the quantification. * *p* < 0.05 for each comparison (*n* = 4); ns: not significant.

SMC phenotypic modulation is characterized by the reduction in contractile proteins. To determine whether YTHDF1 is essential for SMC phenotypic modulation, we tested whether YTHDF1 affects SMC contractile protein expression. Since YTHDF1 is reduced during PDGF-BB-induced SMC phenotypic modulation, we overexpressed YTHDF1 in control and PDGF-BB-treated SMCs. PDGF-BB downregulated endogenous YTHDF1 expression along with a reduction in the SMC contractile proteins ACTA2, CNN1, and TAGLN (Figure 3A–E). However, the overexpression of SNAP-tagged YTHDF1 protein significantly restored the expression of all contractile proteins in PDGF-BB-treated SMCs (Figure 3A–E), indicating that YTHDF1 is a negatively regulator for SMC phenotypic modulation. The overexpression of YTHDF1 also increased the expression of CNN1 and TAGLN expression in PDGF-BB-untreated control SMCs (Figure 3A–E), further suggesting that YTHDF1 promotes a contractile SMC phenotype. Interestingly, we observed that the overexpression of YTHDF1 also increased the endogenous YTHDF1 levels in PDGF-BB-treated SMCs (Figure 3A–E), suggesting that YTHDF1 may negatively affect PDGF-BB signaling through an unidentified mechanism.

### 3.4. YTHDF1 Attenuates Injury-Induced Neointimal Formation/Vascular Remodeling

**Figure 4:**
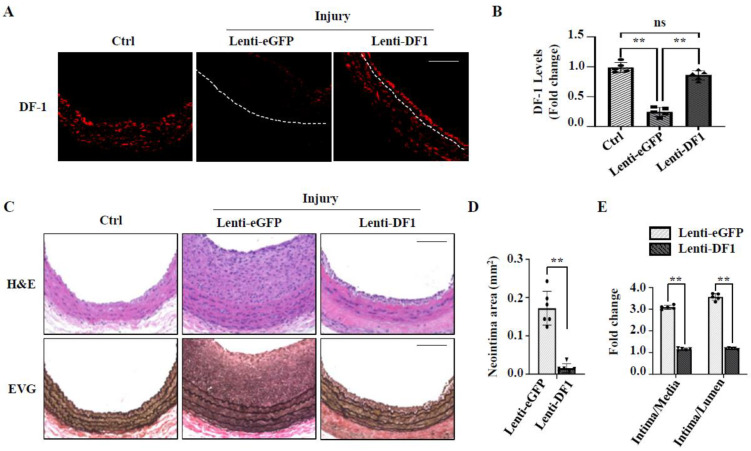
Overexpression of DF1 attenuates injury-induced neointima formation. Rat left common carotid arteries were balloon-injured, and lentiviral vectors expressing GFP (lenti-eGFP) or YTHDF1 (Lenti-DF1) were transduced to the injured sites immediately after the injury. A total of 14 days later, the arteries were collected and paraffin-embedded for histological analyses. Right carotid arteries were used as control (Ctrl). (**A**) Lenti-DF1 delivery restored DF-1 expression in artery that was downregulated by injury, as assessed by immunostaining. Scale bar = 100 μm. (**B**) DF-1 levels were quantified by averaging the fluorescent signal intensity minus the background signal and relative to the signal intensity in the Ctrl images. ** *p* < 0.001 (*n* = 5). (**C**) Ctrl or injured carotid artery sections were stained with H&E (upper panel) and Elastica van Gieson (lower panel). Scale bar = 100 μm. (**D**) Quantification of neointima area. ** *p* < 0.005 (*n* = 6). (**E**) Quantification of intima/media and intima/lumen ratios. ** *p* < 0.01 (*n* = 5). ns: not significant.

To determine whether m6A modification is involved in SMC phenotypic modulation in vivo, we generated a rat carotid artery balloon-injury model because vascular injury induces SMC phenotypic modulation and subsequent neointimal hyperplasia [14,16]. Since YTHDF1 negatively regulated SMC phenotypic modulation in vitro, we first examined YTHDF1 expression during the injury-induced neointimal formation. Immunohistochemistry staining showed that YTHDF1 is highly expressed in media layers of uninjured control carotid arteries (Figure 4A,B). However, balloon injury significantly diminished YTHDF1 expression in the arteries along with dramatic neointima formation (Figure 4A,B), consistent with in vitro observation in PDGF-BB-treated SMCs, suggesting that YTHDF1 may be involved in neointimal formation.

To test whether YTHDF1 is involved in neointimal formation, we transduced a lentiviral vector expressing YTHDF1 (Lenti-DF1) or green fluorescent protein (GFP, control) into the denuded rat common carotid arteries. As shown in Figure 4A,B, although injury blocked YTHDF1 expression in the arteries, Lenti-DF1 transduction successfully restored the YTHDF1 expression in the media and the neointima area of the injured arteries (Figure 4A,B). Importantly, YTHDF1 expression dramatically reduced the injury-caused neointima formation (Figure 4C–E). The intima areas, intima-to-media ratios, and intima-to-lumen ratios were all significantly reduced in Lenti-DF1-transduced arteries as compared to GFP-transduced arteries (Figure 4D,E). These data demonstrate that YTHDF1 is a novel protein factor important for maintaining vascular homeostasis and that a reduction in YTHDF1 contributes to the pathological vascular remodeling.

### 3.5. YTHDF1 Inhibits SMC Phenotypic Modulation In Vivo

**Figure 5:**
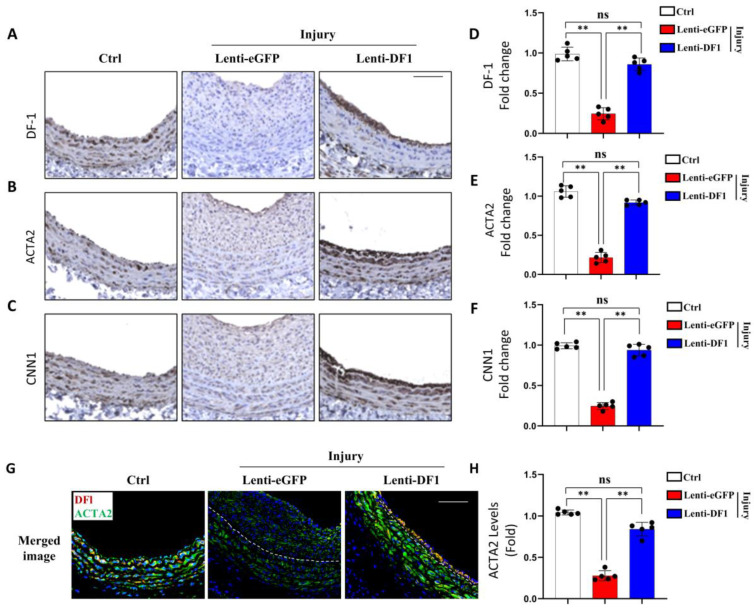
Overexpression of YTHDF1 (DF1) attenuates SMC phenotypic modulation in vivo in injured arteries. (**A**–**C**) Control (Ctrl) and injured rat carotid artery sections with Lenti-GFP or Lenti-DF1 transduction were immunohistochemically stained with DF1 (**A**), smooth muscle α-actin (ACTA2, (**B**)), or Calponin (CNN1, (**C**)) antibody, and the protein levels were visualized by 3,3′-Diaminobenzidine (DAB) staining. Scale bar = 100 μm. (**D**–**F**) Quantification of DF1 (**D**), ACTA2 (**E**), and CNN1 (**F**) by averaging the DAB intensity minus the background signal and relative to the signal intensity in the Ctrl images. ** *p* < 0.001 for each comparison (*n* = 5). (**G**) DF1 co-localized with ACTA2 in SMCs as shown by the co-staining of DF1 with ACTA2 in Ctrl and injured arteries with Lenti-DF1 transduction. Scale bar = 100 μm. (**H**) Quantification of ACTA2 shown in G by averaging the fluorescent signal intensities minus the background and relative to the Ctrl group. ** *p* < 0.001 for each comparison (*n* = 5). ns: not significant.

To determine whether YTHDF1 regulates SMC phenotypic modulation in vivo, we detected the effect of YTHDF1 on SMC contractile protein expression in both control and injured rat carotid arteries by immunohistochemistry staining. As shown in Figure 5, balloon injury downregulated YTHDF1 expression but its expression was significantly elevated by the transduction of the Lenti-DF1 lentiviral vector, especially in the SMC layers. Of importance, although injury significantly downregulated the expression of the contractile proteins ACTA2 and CNN1 in medial SMCs while inducing severe neointima hyperplasia, transduction of YTHDF1 into the arteries resulted in the recovery of ACTA2 and CCN1 expression in artery medias, indicating that YTHDF1 blocked the injury-induced SMC phenotypic modulation in arteries in vivo. To further confirm the role of YTHDF1, we co-stained YTHDF1 and ACTA2 in control and injured arteries with or without Lent-DF1 transduction and found that ACTA2 expression was colocalized with YTHDF1 SMCs. Importantly, the ACTA2 level is correlated with YTHDF1 expression (Figure 5G), further demonstrating that YTHDF1 plays an important role in maintaining contractile SMC phenotype in vivo. The downregulation of YTHDF1 promotes SMC phenotypic modulation and consequently neointimal formation/vascular remodeling.

## 4. Discussion

m6A modification has emerged as an important regulatory mechanism controlling many cellular processes and disease development. Our study indicates that m6A modification also extensively occurs during SMC phenotypic modulation. The m6A modifications of 708 genes were increased while the modifications of 300 genes were decreased, suggesting that m6A modifications are involved in multiple events during the dynamic transformation of contractile to synthetic SMC phenotypes and even the subsequent SMC proliferation and migration. Indeed, mRNAs of genes involved in tissue homeostasis, cell differentiation, cell migration, gene transcription, and a number of cell signaling are modified although their specific roles in SMC phenotypic modulation remain to be determined. Interestingly, m6A writers and erasers are not regulated by PDGF-BB, although m6A modification levels are increased in SMCs. A possible explanation is that other processes such as nuclear translocation of writers or erasers may be altered by PDGF-BB, which requires additional extensive studies in the future. Nonetheless, m6A readers are dramatically altered during SMC phenotype alteration. Among different readers, YTHDF1 appears to be more relevant to SMC phenotypic modulation because its expression was altered before the change in SMC phenotype.

YTHDF1 is a powerful reader for m6A modification, which translationally controls RNA transcripts and participates in many physiological and pathological processes, such as tumor growth and metastasis [17,18], antitumor immune responses [19], axon guidance [20], and the proliferation of native T cells [21]. For cardiovascular diseases, YTHDF1 has been reported to associate with the proliferation [22] and differentiation [23] of cardiomyocytes. In addition, YTHDF1 promotes pulmonary hypertension by modulating the phenotype of pulmonary artery smooth muscle cells [24]. Our study identified YTHDF1 as a novel regulator for SMC phenotypic modulation. YTHDF1 is downregulated along with the reduction in SMC markers in PDGF-treated SMCs. The overexpression of YTHDF1 inhibits the transition of contractile to synthetic SMC phenotype by preserving SMC marker levels.

Importantly, we found that YTHDF1 is able to attenuate intimal hyperplasia in vivo. YTHDF1 is likely to be important for maintaining vascular homeostasis because mechanic injury causes the downregulation of YTHDF1 while inducing intimal hyperplasia. However, when YTHDF1 is stably restored by the transduction of Lenti-YTHDF1, neointima formation was blocked while SMC marker expression was restored. These data indicate that YTHDF1 inhibits intimal hyperplasia by blocking SMC phenotypic modulation in artery media layers. This notion is proved by the correlation of YTHDF1 expression with SMC marker proteins, although lentiviral delivery has a safety concern because random genomic integration could cause unpredictable or detrimental outcomes. Thus, future studies could use new delivery technologies and accurate gene editing methods [25] if targeting YTHDF1 becomes a therapeutic strategy for treating proliferative vascular diseases.

One of the limitations in this study is that it is unclear how YTHDF1 regulates SMC phenotypic modulation. However, since YTHDF1 is downregulated rapidly following PDGF-BB treatment, it is likely to be involved in the regulation of the initial events of SMC phenotypic modulation such as the expression of regulators involved in the transcription or translation of SMC marker genes. In addition, it may also be important to study how PDGF-BB downregulates YTHDF1 in SMCs. Previous studies have shown that several upstream factors can regulate YTHDF1 expression, such as c-Myc [26], the Wnt/β-catenin signaling pathway [27], and miRNAs [28]. Therefore, future studies could determine whether these factors or signaling pathways mediate the PDGF-BB-regulation of YTHDF1. Another limitation is that the study was only conducted in male animals. Female animals need to be included in future studies to examine whether there is a sex difference in m6A modification during SMC phenotypic modulation and whether different m6A regulators are involved in this process in females.

It is worth noting that a previous study showed that YTHDF1 is upregulated by PDGF-BB in airway SMCs, and the overexpression of YTHDF1 promotes rather than inhibits the proliferation and migration of airway SMCs [29]. Another study reported that the inhibition of YTHDF1 prevents hypoxia-induced pulmonary artery SMC proliferation [30]. These discrepancies from our studies are likely due to the different sources of SMCs. It appears that the SMC phenotypes are regulated differently in the lungs than the other system, likely attributed to their different origins during embryonic development, which warrants further investigation.

## 5. Conclusions

Our studies demonstrate that the level of m6A modification changes during SMC phenotypic modulation and injury-induced vascular remodeling. The m6A reader YTHDF1 is an important factor maintaining contractile SMC phenotype and vascular homeostasis. Delivering YTHDF1 can efficiently attenuate injury-induced neointima. Therefore, the restoration of YTHDF1 may be a promising strategy for preventing the development of surgery-induced vascular remodeling and other proliferative vascular diseases.

## Data Availability

The original contributions presented in this study are included in the article/Supplementary Material. Further inquiries can be directed to the corresponding author.

## Supplementary Materials

The following supporting information can be downloaded at: https://www.mdpi.com/article/10.3390/cells14030160/s1, Original Images for Blots.
