# Supplementary figures and images for: YTH N6-methyladenosine RNA Binding Protein 1 Inhibits Smooth Muscle Cell Phenotypic Modulation and Neointimal Hyperplasia
Source: Cells. 2025 Jan 22;14(3):160. doi: 10.3390/cells14030160 (PMC11817666; doi:10.3390/cells14030160)

# Original Images for Blots

Figure 2A

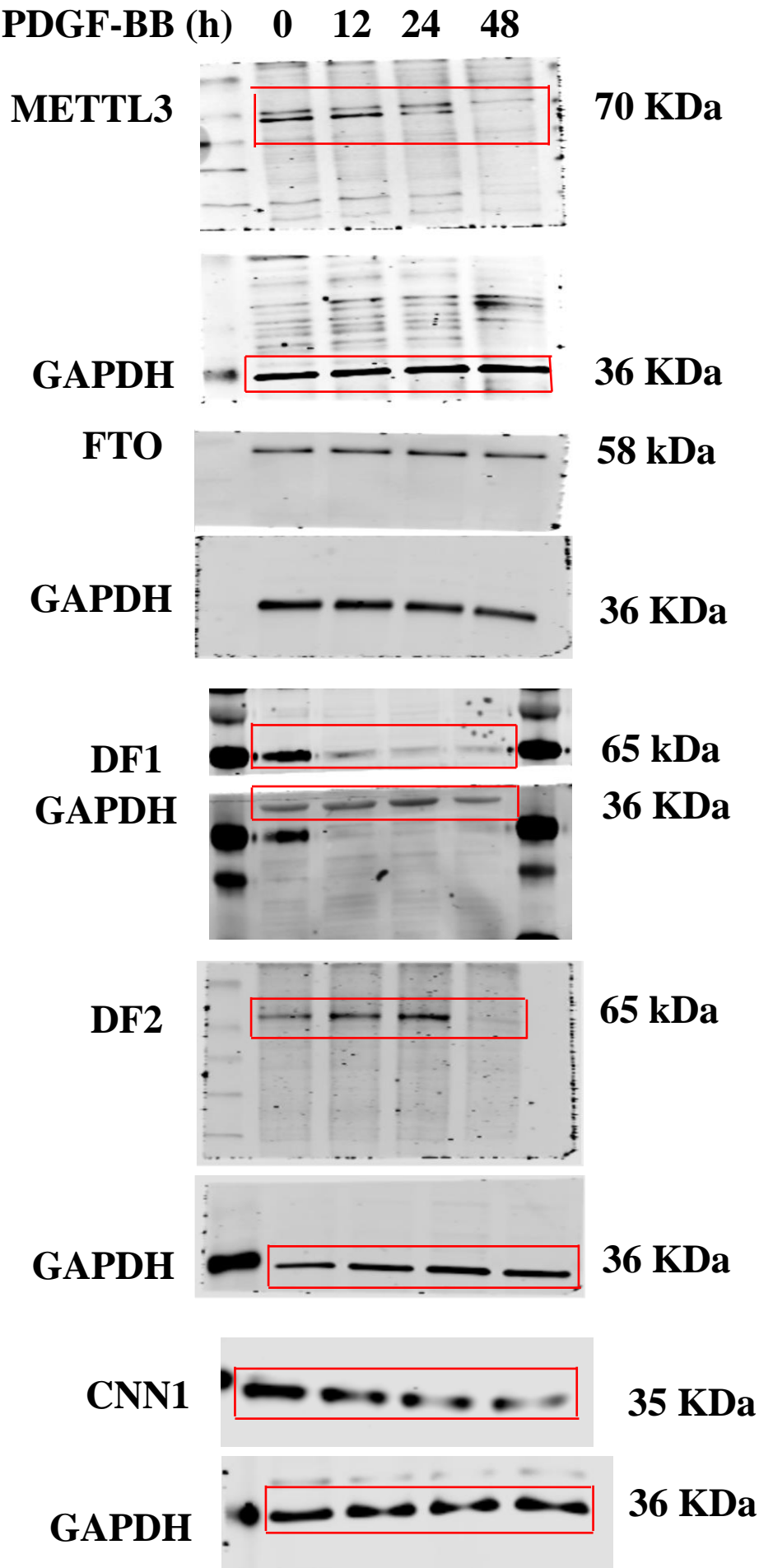

# Original Images for Blots

Figure 3A

|         |   |   |   |   |
|---------|---|---|---|---|
| Control | + | - | + | - |
| DF1     | - | + | - | + |
| PDGF-BB | - | - | + | + |

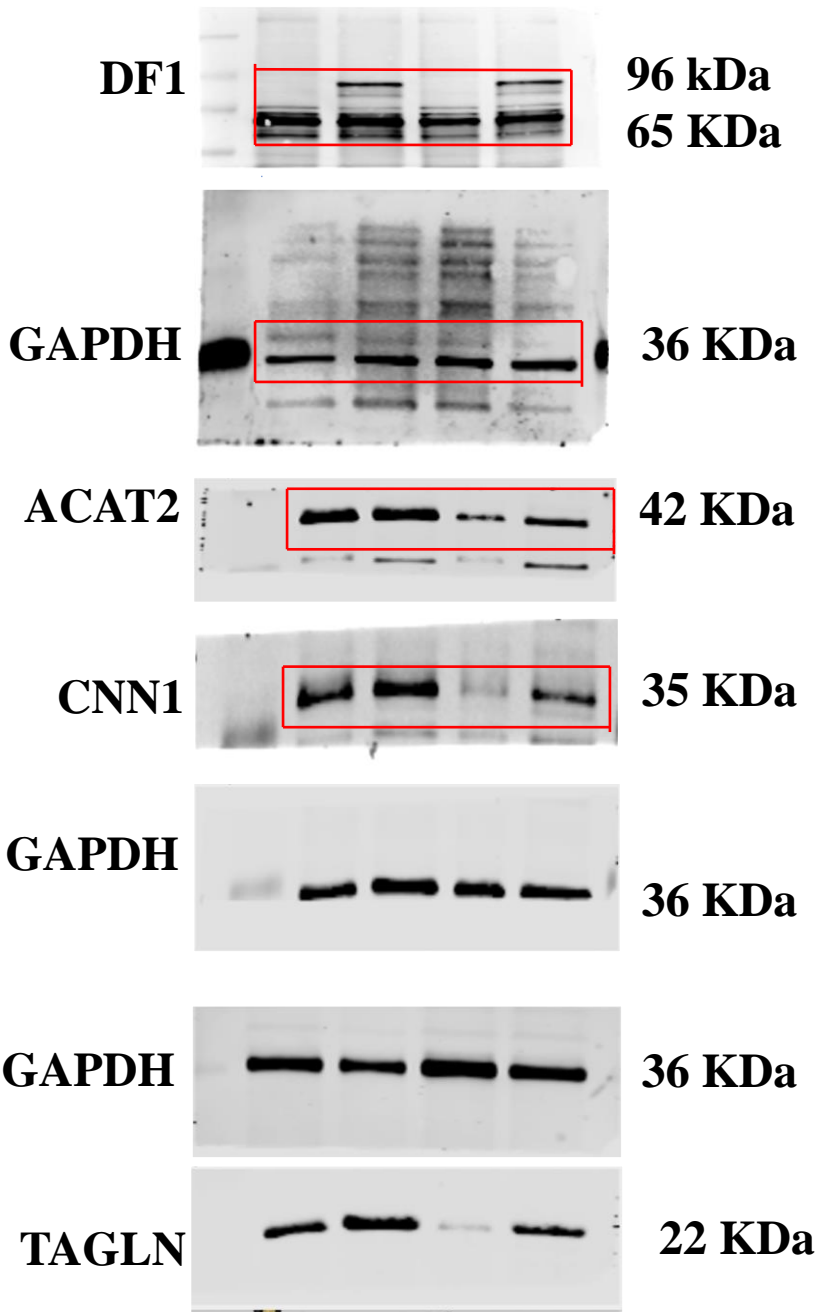

Supplement: Supplementary file 1 [file cells-14-00160-s001.zip › cells-3279453-supplementary.pdf]
